# Rbfox2 function in RNA metabolism is impaired in hypoplastic left heart syndrome patient hearts

**DOI:** 10.1038/srep30896

**Published:** 2016-08-03

**Authors:** Sunil K. Verma, Vaibhav Deshmukh, Curtis A. Nutter, Elizabeth Jaworski, Wenhao Jin, Lalita Wadhwa, Joshua Abata, Marco Ricci, Joy Lincoln, James F. Martin, Gene W. Yeo, Muge N. Kuyumcu-Martinez

**Affiliations:** 1 Department of Biochemistry and Molecular Biology, University of Texas Medical Branch, Galveston, Texas-77555, USA; 2 Department of Molecular Physiology and Biophysics, Baylor College of Medicine, Houston, TX 77030, USA; 3 Department of Physiology, National University of Singapore, Singapore 117597; 4 Division of Congenital Heart Surgery, Baylor College of Medicine, Houston, TX, 77030, USA; 5 Pensacola Christian College, 250 Brent Ln, Pensacola, FL 32503, USA; 6 Department of Surgery, University of New Mexico College of Medicine, Albuquerque, NM, 87131, USA; 7 Center for Cardiovascular Research and The Heart Center, Nationwide Children’s Hospital Research Institute, The Ohio State University, Columbus, OH 43205, USA; 8 Department of Pediatrics, The Ohio State University, Columbus, OH 43205, USA; 9 Program in Developmental Biology, Division of Cardiology, Baylor College of Medicine; Texas Heart Institute, Houston, TX 77030, USA; 10 Department of Cellular and Molecular Medicine, Stem Cell Program and Institute for Genomic Medicine University of California, San Diego, La Jolla, CA 92093, USA; 11 Department of Neuroscience and Cell Biology, University of Texas Medical Branch, Galveston, Texas-77555, USA; 12 Institute for Translational Sciences, University of Texas Medical Branch, Galveston, Texas-77555, USA

## Abstract

Hypoplastic left heart syndrome (HLHS) is a fatal congenital heart disease in which the left side of the heart is underdeveloped, impairing the systemic circulation. Underdeveloped left ventricle exerts biomechanical stress on the right ventricle that can progress into heart failure. Genome-wide transcriptome changes have been identified at early stages in the right ventricle (RV) of infants with HLHS, although the molecular mechanisms remain unknown. Here, we demonstrate that the RNA binding protein Rbfox2, which is mutated in HLHS patients, is a contributor to transcriptome changes in HLHS patient RVs. Our results indicate that majority of transcripts differentially expressed in HLHS patient hearts have validated Rbfox2 binding sites. We show that Rbfox2 regulates mRNA levels of targets with 3’UTR binding sites contributing to aberrant gene expression in HLHS patients. Strikingly, the Rbfox2 nonsense mutation identified in HLHS patients truncates the protein, impairs its subcellular distribution and adversely affects its function in RNA metabolism. Overall, our findings uncover a novel role for Rbfox2 in controlling transcriptome in HLHS.

Hypoplastic left heart syndrome (HLHS) is one of the least understood, fatal congenital heart diseases that affects 1 in 4,344 newborns^1^,^2^. Infants with HLHS suffer from varying degrees of hypoplasia of the left ventricle (LV), left atrium (LA), and ascending aorta, closing or narrowing of mitral, and aortic valves^3^. HLHS patients need three stages of surgery or cardiac transplantation to reestablish the systemic circulation^3^. Even post-surgery, patients are at high risk to develop right ventricle (RV) failure and/or abnormal heart rhythm due to hypoxic conditions, pressure and volume overload^1^,^4^,^5^,^6^. Mechanisms leading to the progression of RV overload into RV failure are largely unknown. Aberrant alternative splicing (AS) patterns and mRNA levels have been identified in HLHS RVs obtained at early stages during Stage 1 Norwood surgery^5^. Identification of early changes in HLHS RV will help better understand the progression of RV overload into heart failure and also help determine novel biomarkers for RV failure in HLHS patients.

Although genome-wide gene expression changes were identified in HLHS patient RVs^5^, the molecular events responsible for aberrant transcriptome in HLHS patients are unclear. Therefore, there is a pressing need to determine such mechanisms to better understand HLHS RV pathogenesis and identify novel targets for potential therapy options.

The RNA binding protein Rbfox2 has been recently identified as a major risk allele for HLHS^7^. In a large cohort of congenital heart disease patients, three *de novo* detrimental Rbfox2 mutations significantly associated with HLHS phenotype^7^. Rbfox2 belongs to a family of RNA-binding proteins with affinity for (U)GCAUG rich sequences, which are highly conserved among vertebrates in both sequence and position^8^,^9^. Rbfox2 binding targets were identified in mouse and human ES cells and 293T cells using cross-linking immunoprecipitation followed by RNA-sequencing (CLIP-seq) analysis^10^,^11^,^12^. Rbfox2 controls alternative splicing (AS) events critical for the survival of ES cells, differentiation of pluripotent stem cells^13^ and during epithelial-mesenchymal transition^14^,^15^. Conditional deletion of Rbfox2 in the cerebellum leads to AS and developmental defects^16^. Knockdown of both Rbfox2 and its paralog Rbfox1 in zebrafish lowers heart rate and causes myofibrillar disarray^17^. Downregulation of Rbfox2 is associated with pressure overload induced heart failure in mice^18^. Our results indicate that Rbfox2 is also involved in diabetic heart disease^19^; suggesting an important role for Rbfox2 in human heart diseases.

In this study, we provide evidence that Rbfox2 has a role in HLHS pathogenesis. We demonstrate that HLHS patient specific mutation in Rbfox2 induces a dramatic change in Rbfox2 protein subcellular localization and affects its function in RNA splicing. In the future, our findings may allow identification of innovative therapy strategies such as the use of modified oligos or small molecules to restore Rbfox2 function in patients with HLHS.

## Results

### Rbfox2 undergoes changes in HLHS patients’ right ventricles

**Figure 1:**
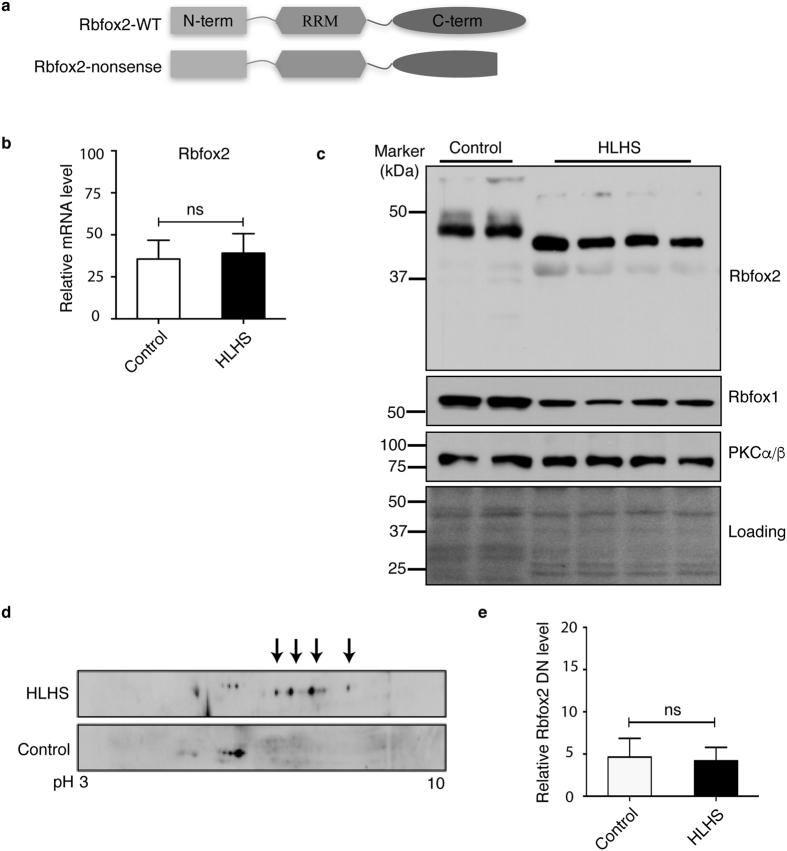
Rbfox2 undergoes changes in HLHS patient hearts. (**a**) The effect of *de novo* nonsense mutation identified in HLHS patients on Rbfox2 protein. (**b**) Quantification of mRNA levels of Rbfox2 in control and HLHS patient hearts determined by qRT-PCR after normalizing to GADPH mRNA levels. (n ≥ 3), ns: not significant. (**c**) WB analysis of heart protein lysates from controls or HLHS patients using anti-Rbfox2, anti-Rbfox1 and anti-PKCalpha/beta antibodies. Ponceau S staining of the membrane was used to monitor protein loading. (**d**) Analysis of Rbfox2 protein in control or HLHS patient hearts using two-dimensional (2D) gel electrophoresis followed by WB using anti-Rbfox2 antibody. Four black arrows indicate Rbfox2 isoforms in HLHS hearts. (**e**) DN Rbfox2 expression in control vs HLHS patient hearts determined by exon 6 exclusion using qRT-PCR. (n ≥ 3), ns: not significant.

To determine regulators that account for abnormal gene expression in HLHS patient RVs, we focused on the RNA binding protein Rbfox2, because it was identified as a major risk allele for HLHS using a large cohort of congenital heart disease patients^7^. Rbfox2 mutations were significantly associated with HLHS among other congenital heart defects^7^. HLHS specific Rbfox2 mutations (splice site, nonsense and frameshift) are predicted to either target Rbfox2 mRNA for degradation (data not shown) or truncate the protein removing a portion of the C-terminal domain (CTD) of the protein (Fig. 1a and Supplementary Fig. 3). Splice site mutation is expected to cause inclusion of ~1.6 kb intron 10 and likely degradation of Rbfox2 mRNA by nonsense-mediated decay (NMD). Frameshift mutation is also predicted to introduce a stop codon immediately after the mutation potentially leading to NMD. Nonsense Rbfox2 mutation is expected to remove a small portion of the CTD (Fig. 1a and Supplementary Fig. 3). Notably, the CTD of Rbfox2 is necessary for interactions of Rbfox2 with several RNA binding proteins and spliceosome component U1C and localization to the nucleus^20^,^21^,^22^.

We first checked Rbfox2 mRNA levels in HLHS patient hearts by real time qRT-PCR using gene specific primers (Supplementary Table 2). No change in mRNA levels of Rbfox2 was identified in HLHS patient hearts in comparison to the controls (Fig. 1b). To investigate if there are any changes in protein levels, we analyzed protein lysates from human HLHS patient and control hearts by Western blot (WB) using commercially available Rbfox2 antibody. A striking ~3KDa change in MW of Rbfox2 protein was observed in HLHS patient hearts in comparison to control hearts (Fig. 1c). We also tested whether Rbfox1, a family member of Rbfox2, is affected in HLHS patient hearts. Rbfox1 is expressed and dynamically regulated in developing postnatal hearts^23^. Rbfox1 protein was modestly downregulated (1.5 fold change) in HLHS patient hearts without a change in its MW (Fig. 1c). To further investigate the change in MW of Rbfox2, we assessed Rbfox2 protein pattern on 2-dimensional (2D) gels followed by WB. 2D/WB analysis of Rbfox2 indicates that not only the MW but also the isoelectric point (pI) of Rbfox2 was altered in HLHS patient hearts. Control human heart lysates displayed three Rbfox2 specific spots with a pI of ~6 (Fig. 1d, bottom panel). These spots were barely detectable in HLHS hearts (Fig. 1d, top panel). Strikingly, four spots with lower molecular weight and more basic pI (pH ~7 to 8) were predominant in HLHS patient heart lysates (Fig. 1d, top panel; marked with arrows). Proteins can become basic pI when they lose post-translational modifications such as phosphorylation. We have shown that Rbfox2 is regulated in a PKC-dependent manner^24^. Thus, we tested whether PKCα/β is altered in HLHS patients that could explain changes in Rbfox2 protein levels. There was no obvious change in PKCα/β protein levels or activation status in HLHS patient hearts (Fig. 1c and data not shown). These results suggest that changes in Rbfox2 in HLHS patient hearts are independent of PKCα/β.

Since Rbfox2 has several spliced variants including a dominant negative (DN) isoform that has a lower molecular weight due to the exclusion of exon 6, which encodes a large portion of the RNA recognition domain^25^, we tested whether the lower migrating isoform of Rbfox2 protein is the DN isoform. DN Rbfox2 expression was determined by qRT-PCR in HLHS patient hearts using primers to detect exon 6 inclusion (Supplementary Table 2). Figure 1e shows that there was no difference in Rbfox2 DN expression level in HLHS patients versus control hearts indicating that the low MW isoform present in HLHS patients is not the DN isoform of Rbfox2.

### Subcellular distribution of Rbfox2 protein is altered in HLHS patient right ventricles

**Figure 2:**
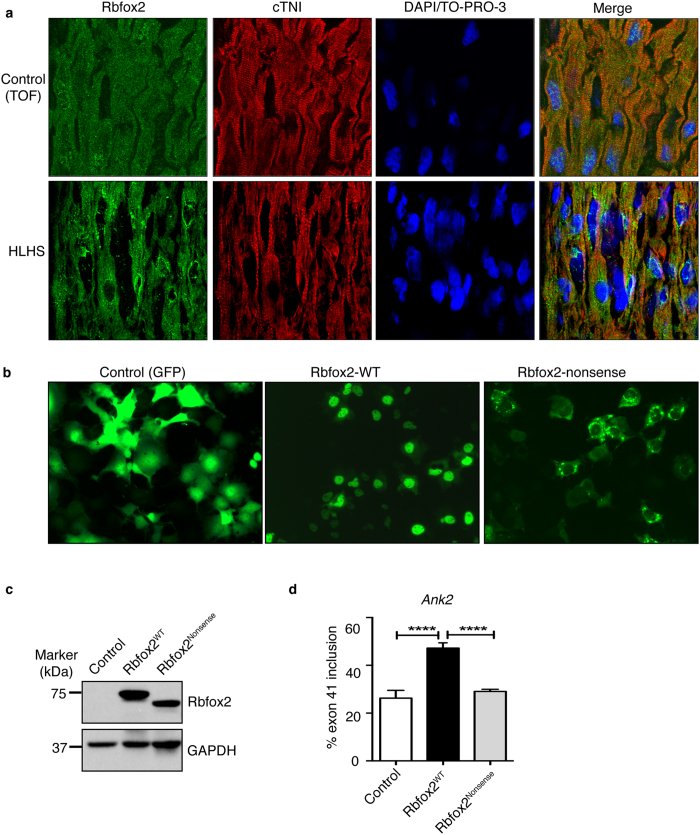
Subcellular distribution of Rbfox2 is altered in HLHS right ventricles. (**a**) Immunofluorescence (IF) of Rbfox2 in right ventricle sagittal sections of infants with HLHS or Tetralogy of Fallot (control). Cardiomyocytes were marked with cardiac troponin I. Nuclei were stained with DAPI/TO-PRO-3. Fluorescence images were obtained at 80X magnifications with a confocal laser-scanning microscope (LSM 510META, Carl Zeiss) at the University of Texas Medical Branch imaging core facility. (**b**) Representative images of GFP (control), GFP-tagged wild type (Rbfox2^WT^) and nonsense mutant (Rbfox2^Nonsense^) of Rbfox2 protein in COS M6 cells. (**c**) WB analysis of GFP-Rbfox2^WT^ and GFP-Rbfox2^Nonsense^ protein in COS M6 cells using anti-Rbfox2 antibody. GAPDH WB was used as a loading control. (**d**) Percent inclusion of *Ank2* exon 41 in control (vector), GFP-Rbfox2^WT^ or Rbfox2^Nonsense^ mutant expressing COS M6 cells determined by qRT-PCR (n ≥ 3). Each reaction was done as triplicates for 3 different experiments and significance was calculated using one-way ANOVA.

Since Rbfox2 HLHS specific nonsense mutation is expected to truncate the protein potentially affecting its nuclear localization, we checked Rbfox2 subcellular distribution in HLHS patient RVs. Neonatal patient hearts with Tetratology of Fallot (TOF) were used as controls for evaluating HLHS specific changes independent of hemodynamic effects on systemic RV dysfunction. Rat newborn and adult, mouse embryonic and human adult hearts were also used as additional controls for determining Rbfox2 localization because of the difficulty in obtaining heart biopsy samples from healthy infants. DAPI/TO-PRO-3 was used to mark the nucleus and cardiac troponin I to mark cardiomyocytes (Fig. 2a). Using frozen heart sections, we found that Rbfox2 was localized both in the nucleus and cytoplasm of cardiomyocytes in control RVs obtained from infants with TOF (Fig. 2a, top panel). In contrast, Rbfox2 was predominantly cytoplasmic in punctate bodies with barely detectable levels in the nucleus of cardiomyocytes in HLHS patient RVs (Fig. 2a, lower panel). Rbfox2 was localized to both nucleus and cytoplasm in rat (newborn and adult), mouse (embryonic) and human (adult) hearts but more prominently in the nucleus of rat newborn and adult hearts and embryonic mouse hearts (Supplementary Fig. 2). These results combined with our observation on WB and 2D/WB indicate that Rbfox2 protein displayed a lower MW and localized predominantly in the cytoplasm of HLHS patient RVs in contrast to the pattern in control RVs.

To test the effect of HLHS specific mutations on Rbfox2 expression, localization and function, we generated a nonsense mutant of GFP-tagged Rbfox2 based on the mutations identified in HLHS patients. We expressed either wild type (WT) or HLHS specific nonsense mutant of GFP-Rbfox2 in COS M6 cells. WT Rbfox2 was mostly nuclear whereas HLHS specific Rbfox2 nonsense mutant was predominantly cytoplasmic in punctate bodies around the nuclear envelope resembling its pattern seen in HLHS patient hearts (Fig. 2b *vs*. 2a). HLHS specific nonsense Rbfox2 mutant displayed a lower MW (Fig. 2c) as predicted based on the mutation site (Fig. 1a and Supplementary Fig. 3). Nonsense mutant of Rbfox2, which lacks a portion of the CTD that has a nuclear localization signal and binding sites for splicing regulators, was unable to control alternative splicing of its target *Ank2* as efficiently as WT Rbfox2 (Fig. 2d). In summary, HLHS patient specific nonsense mutation affects the subcellular localization of Rbfox2 impacting its nuclear function in RNA splicing.

### A significant number of transcripts altered in HLHS patient hearts are Rbfox2 targets

**Figure 3:**
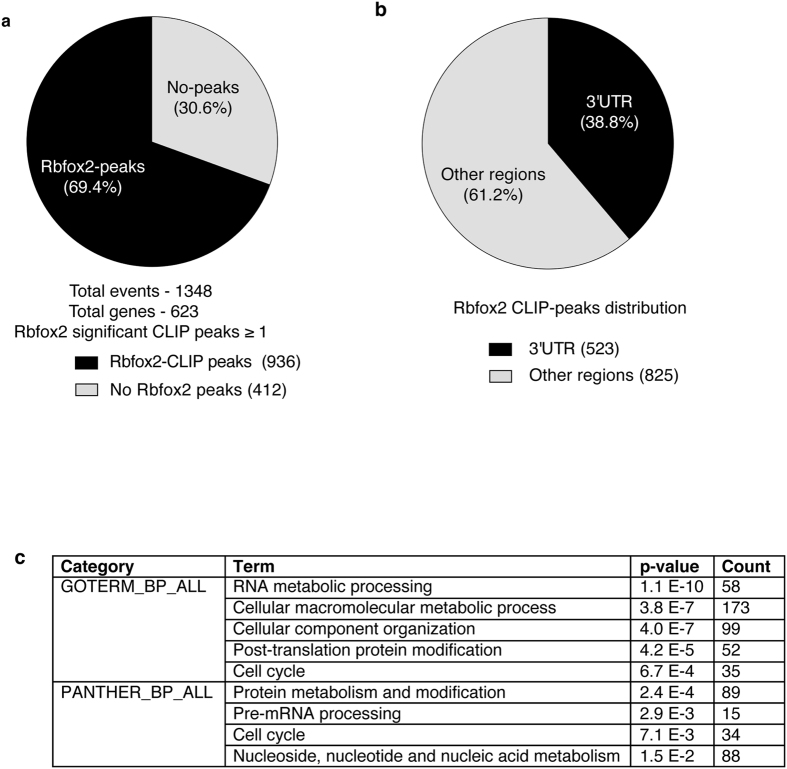
A large group of transcripts altered in HLHS patient hearts have Rbfox2 binding sites. (**a**) Pie chart of Rbfox2 binding sites on 1348 transcripts that are differentially expressed in HLHS patients. (**b**) Rbfox2 binding site distribution on transcripts differentially expressed in HLHS patient hearts. (**c**) Top GO categories of genes with Rbfox2 binding sites.

Because Rbfox2 protein was mostly cytoplasmic in HLHS patient RVs, we tested whether transcripts differentially expressed in HLHS RVs are Rbfox2 targets. We used HLHS patient exon array data to extract genomic locations for differentially expressed transcripts in HLHS patient hearts. Using the available HLHS patient exon array data, we extracted sequences for differentially expressed genes in HLHS patient hearts. These sequences were mapped to the human genome to access Rbfox2 CLIP-binding density information^10^. Rbfox2 CLIP-seq analysis was performed in human ESCs and Rbfox2 binding clusters were found to be statistically significant in 85% of the binding sites identified when compared to the rest of the genome^10^. Our analysis of 1348 transcripts (623 genes) that are differentially expressed in HLHS patients revealed that 69.4% of the transcripts contain statistically significant Rbfox2 CLIP-peaks (Fig. 3a). We used genes that are not significantly affected in HLHS patients as controls for this analysis. Consistent with its function in AS regulation, 934 transcripts exhibited significant Rbfox2-CLIP clusters in intronic and/or exonic regions of the differentially expressed transcripts (Supplementary Fig. 1). Rbfox2 binding sites were mostly enriched in the 3′ untranslated region (UTR) of 38.8% of the total transcripts analyzed (Fig. 3b). When genes with Rbfox2 binding sites were categorized based on their biological function using DAVID server (whole genome was used as a background control), the top statistically significant groups were RNA/protein metabolic processes and cell cycle in agreement with the previous findings^10^,^16^ (Fig. 3c). Overall, these results indicate that a large portion of transcripts modulated in HLHS patients are previously identified Rbfox2 targets that have important roles in cell cycle and RNA/protein metabolism.

### Rbfox2 controls mRNA levels that are affected in HLHS patients’ right ventricles

**Figure 4:**
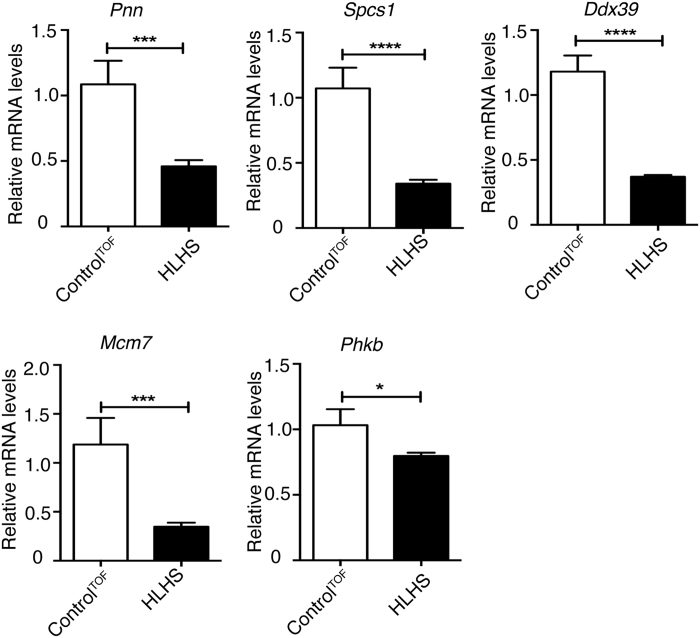
Transcripts with Rbfox2 binding sites are dysregulated in HLHS patient right ventricles. Real time qRT-PCR analysis of *Pnn, Spcs1, Ddx39, Mcm7* and *Phkb* mRNA levels in right ventricles of infants with TOF (control, white bars) or infants with HLHS (black bars). Each mRNA levels were normalized to the internal control (*Polr2a*). Each reaction was done as triplicates and significance was calculated by unpaired t-test (n > 3).

**Figure 5:**
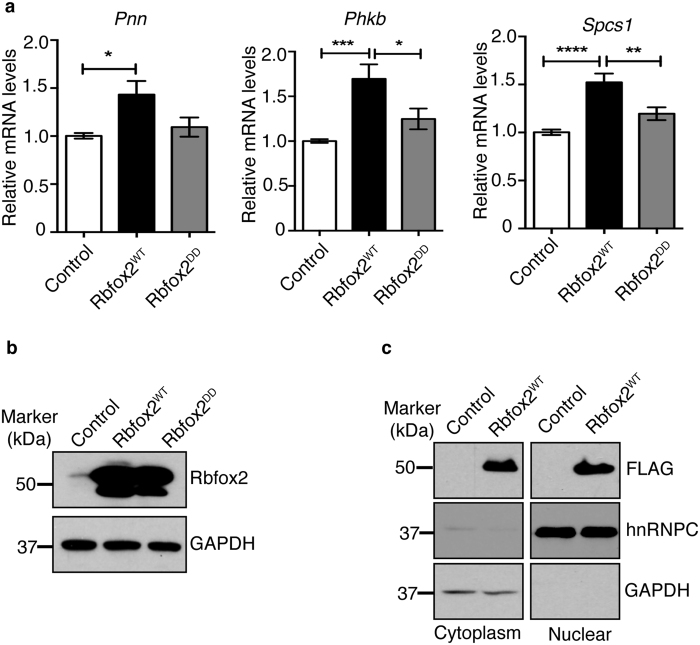
Rbfox2 regulates mRNA levels of target genes dysregulated in HLHS. (**a**) Real time qRT-PCR analysis of *Pnn*, *Phkb*, and *Spcs1* mRNA levels in control (vector), Rbfox2^WT^ or Rbfox2^DD^ expressing COS M6 cells. Results are representative of three independent experiments. Significance was calculated using one-way ANOVA (n > 3). (**b**) WB analysis of Rbfox2 in COS M6 cells. GADPH WB was used to monitor protein loading. (**c**) WB analysis of Flag-Rbfox2^WT^ in nuclear and cytoplasmic fractions of COS M6 cells using anti-FLAG antibody. GADPH WB was used as cytoplasmic marker, and hnRNPC WB as a nuclear marker.

To validate transcriptome changes, we used HLHS patient RV biopsy samples obtained at early stages during Stage 1 surgery. HLHS patients LVs, which are severely underdeveloped and undergone decompensation, were excluded for our analyses. Using RNA from RV heart biopsy samples, we validated mRNA levels of 5 genes (*Pnn, Spcs1, Ddx39, Mcm7, Phkb*) that were identified as the top changers in HLHS patient RV transcriptome analysis^5^. Functions of these genes are described in Supplementary Table 1. Importantly, all 5 mRNAs displayed Rbfox2 binding sites in their 3′UTRs. For real time RT-PCR analysis, 3 different internal controls with similar results were obtained, so the results from one of the internal controls (DNA-directed RNA polymerase II subunit RPB1 (*Polr2a*)) were shown in Fig. 4. *Pnn, Spcs1, Ddx39, Mcm7* and *Phkb* mRNAs were downregulated in HLHS patient RVs when compared to control RVs obtained from Tetralogy of Fallot patients (Fig. 4, black bar *vs* white bar). To determine if Rbfox2 regulates mRNA levels of putative targets altered in HLHS, we ectopically expressed Flag tagged wild type Rbfox2 (Rbfox2^WT^) or RNA binding deficient mutant (Rbfox2^DD^). We chose COS M6 cells for this study, as endogenous Rbfox2 levels are very low in COS M6 cells and should not interfere with the overexpressed Rbfox2 proteins. Expression of Flag-Rbfox2^WT^ increased mRNA levels of *Pnn*, *Phkb*, and *Spcs1* significantly (Fig. 5a; white *vs* black bars). Expression of the RNA binding deficient mutant of Rbfox2 (Rbfox2^DD^) did not affect mRNA levels of *Pnn, Phkb* and *Spcs1* demonstrating that Rbfox2 binding ability is necessary to regulate mRNA levels of these putative targets (Fig. 5a; white *vs* gray bars). *Ddx39* mRNA levels were undetectable in these cells. *Mcm7* mRNA levels were unaffected in Rbfox2 expressing cells suggesting that change in *Mcm7* mRNA levels in HLHS patients are independent of Rbfox2 (data not shown). Rbfox2^DD^ was generated by mutating 2 critical phenylalanine residues in the RNA recognition motif essential for RNA binding activity^26^,^27^,^28^. Rbfox2^WT^ and Rbfox2^DD^ mutant proteins were expressed at similar levels in these cells (Fig. 5b). Importantly, Flag-Rbfox2^WT^ protein was not only nuclear but also cytoplasmic (Fig. 5c) consistent with an increase in mRNA levels of its putative targets (Fig. 5a). Cytoplasmic fractions were free from nuclear contamination as hnRNPC was only present in the nuclear fraction (Fig. 5c). These findings indicate that Rbfox2 controls mRNA levels of targets with 3′UTR binding sites and RNA-binding activity of Rbfox2 is necessary for regulation of mRNA levels. Overall, we find that Rbfox2 protein localization and function is disrupted in HLHS.

## Discussion

HLHS is one of the most complex and challenging congenital heart diseases. In HLHS patients, the left side of the heart cannot support blood flow to the organs due to severe hypoplasia. Without surgical intervention and/or cardiac transplantation, HLHS can be fatal within the first hours or days after birth^1^,^2^. Post-surgery, the RV takes over the LV function^3^. Pressure and volume overload on the RV can lead to heart failure and arrhythmias at post-natal stages^3^,^6^. Mutations in certain genes are identified in HLHS patient tissues^29^,^30^,^31^,^32^,^33^, yet underlying genetic, molecular and cellular events responsible for HLHS RV pathogenesis are not well understood. Detrimental Rbfox2 mutations have been identified and significantly associated with HLHS phenotype in a recent large human genetic study^7^. Our studies show that Rbfox2 nonsense mutation identified in HLHS patients alters proper localization of Rbfox2 in the cell and adversely affects its role in RNA metabolism contributing to early transcriptome changes in HLHS patients. Here, we show that 936 differentially expressed transcripts identified in the RV of infants with HLHS have Rbfox2 binding sites (Fig. 3).

We found that Rbfox2 that is predominantly localized to the cytoplasm in HLHS patient hearts (Fig. 2a). Rbfox2 has many isoforms generated through alternative promoter usage and AS^25^,^34^, especially a dominant negative isoform that lacks RNA binding activity and possesses inhibitory splicing activity^25^. Our findings demonstrate that Rbfox2 isoform expressed in HLHS is not the dominant negative version with inhibitory splicing function (Fig. 1e). We find that Rbfox2 has a different pI in HLHS patient hearts (Fig. 1d). We show that HLHS specific nonsense mutation truncates Rbfox2 protein at the CTD modulating its cellular distribution and function similar to its pattern observed in HLHS patient RVs (Fig. 2b,d).

Our results provide new insights into HLHS pathogenesis by identifying Rbfox2 as a contributor to gene expression changes in the RV of HLHS patients. By integrating Rbfox2 CLIP-sequencing and HLHS patient transcriptome data, we determined Rbfox2 binding sites on 69% of 1348 transcripts that undergo changes in HLHS patient RVs and 38.8% of these transcripts have binding sites within 3′UTRs. We found that Rbfox2 regulated genes have functions in cell cycle and macromolecular metabolism (Fig. 3). We tested mRNA levels of five putative Rbfox2 targets with Rbfox2 binding sites in their 3′UTRs because they were altered the most in HLHS patient RVs. These target genes are implicated in regulating cell proliferation, differentiation, and apoptosis, processes thought to be dysregulated in HLHS. *Pnn* encodes for pinin protein that plays a crucial role in small intestinal development by influencing epithelial cell differentiation^35^. *Phkb* encodes for glycogen phosphorylase kinase that is important for glycogen breakdown and cell growth^36^. Ddx39 is an RNA helicase that regulates the switch between cellular proliferation and differentiation in Xenopus^37^. MCM7 is important for DNA replication and cell growth^38^. These findings suggest that Rbfox2 contributes to cardiac transcriptome changes in HLHS patients by affecting mRNA levels of genes involved in cell cycle and metabolism.

While Rbfox2 function in AS is well-characterized, its function in other aspects of mRNA metabolism is largely unknown. Our results reveal a new role for Rbfox2 in controlling mRNA levels in the heart. In support of our findings, Rbfox1, one of the Rbfox family members, has recently been found to regulate mRNA stability and translation in the brain^39^. Our data show that there is only modest downregulation of Rbfox1 protein in HLHS patient RVs. In summary, our results demonstrate that Rbfox2 nonsense mutation identified in HLHS patients alters its localization and function contributing to aberrant transcriptome changes of its targets in HLHS patients.

## Methods

### Rbfox2 CLIP cluster identification on transcripts altered in HLHS patients

Genomic sequences for 1348 probe locations^5^ were extracted using the online Galaxy ‘Fetch sequence’ tool with the criterion that the sequence length is between 20 nt and 2.5 kb. Each genomic location was extended by 500 nt upstream and downstream before mapping Rbfox2 CLIP-clusters obtained from human ESC cells^10^. Each of the sequences were further analyzed and categorized based on statistically significant CLIP-clusters present in exon, intron and UTR regions of RNAs.

### Cell culture and transfections

COS M6 cells were cultured and maintained as described previously^24^. COS M6 cells (1.0 × 10^6^) were transfected using FuGENE 6 transfection reagent (Promega E2692) and 1 μg of Flag or GFP tagged wild type (Rbfox2^WT^) or RNA binding deficient mutant (Rbfox2^DD^) or nonsense mutant of Rbfox2 (Rbfox2^Nonsense^) plasmid DNA according to the manufacturer’s protocol. Corresponding vector DNAs were used as negative controls. Cells were harvested 72 hrs post-transfection for RNA or protein extraction as described previously^24^.

### RNA Extraction

RNA was extracted from cells and human heart tissues using TRIzol (Invitrogen) according to the manufacturer’s protocol. RNA concentrations were measured using EPOCH Microplate Spectrophotometer (BioTek) and quality of the RNA was assessed using the bioanalyzer at the University of Texas Medical Branch Next Generation Sequencing Core Facility.

### Splicing analysis

The qRT-PCR was performed using a previously described protocol^24^. Primer sequences were designed to detect inclusion/exclusion of alternative exons of *Ank2* (Supplementary Table 2). For all genes, PCR was carried out using 5 μl cDNA and 100ng primer pair with Biolase Taq polymerase (Bioline BIO-21042) using following amplification conditions: 95 °C 45 s; 59 °C 45 s; 72 °C 1 min for 24 cycles. PCR products were resolved on 5% non-denaturing polyacrylamide gels, stained with ethidium bromide and imaged using the Biorad XR imaging system. Unsaturated band intensities were quantified and percent exon inclusion was calculated^24^. All amplified DNA bands were gel isolated and sequenced.

### Real time RT-PCR

The cDNA was synthesized from 2.0 μg RNA using Biolase DNA polymerase (Bioline) with random OligodT primers (Invitrogen) as detailed previously^24^. cDNA was diluted fifty times and used for real time qPCR reaction using LightCycler^®^ 480 SYBR Green I Master mix (Roche) and Roche LightCycler 480 (Roche diagnostic). Specific primers for qPCR were designed to detect the total mRNA levels for *Pnn, Spcs1, Ddx39, Mcm7*, and *Phkb* genes (Supplementary Table 2). *Polr2a* was used as internal control to normalize the mRNA levels. Relative mRNA level in comparison to controls were determined using 2^−ΔΔct^ method.

### 2 dimensional (2D) gels and Western blotting

2D gel electrophoresis was performed as described previously^24^. Proteins (30–50 μg/sample) were separated by isoelectric focusing using strips that range from pH 3 to 10, then loaded on 10% SDS-PAGE gels, and transferred to a PVDF membrane. Membranes were blocked with 5% dry fat-free milk solution in PBST (PBS containing 0.1% Tween 20) and incubated overnight at 4 °C with the indicated antibodies: Rbfox2 (abcam #57154; 1:500), GAPDH (abcam #9484; 1:2500). Membranes were washed with PBST 3 × 15 minutes and incubated with HRP-labeled secondary antibody for 2 hrs at room temperature. HRP activity was determined using Immobilon Western Chemiluminescent reagent (Millipore P90720) or SuperSignal West Femto Chemiluminescent (Pierce PI34095) HRP substrate followed by exposure to x-ray film.

### Rbfox2 plasmid constructs

Human Flag-tagged Rbfox2 cDNA clone (transcript variant 6) was generously provided by Dr. Douglas L. Black (UCLA). An Rbfox2 mutant lacking RNA binding activity (Rbfox2^DD^) was generated by mutating two critical Phenylalanine 153 and 155 residues into aspartic acid residues necessary for RNA binding using Quick-change site directed mutagenesis kit (Agilent Technologies Inc.)^26^,^27^,^28^. Human GFP-tagged Rbfox2 cDNA clone (transcript variant 3) was obtained form Addgene plasmid repository (plasmid # 63086). Rbfox2 nonsense mutant was generated by replacing nucleotide C to T at equivalent chromosomal position (Ch22:36,155,972)^7^ in Flag and GFP tagged Rbfox2 DNA using site directed mutagenesis.

### Statistics

Data are represented as mean ± SEM or ±SD. Data from at least three independent experiments were used for statistical analysis, and significance was calculated using an unpaired t-test for two group comparisons or one-way ANOVA with Bonferroni correction for three and four different group comparisons using GraphPad Prism software.

### Human Heart tissues

All human heart tissues were de-identified and pre-existing. Human heart RV biopsy samples were obtained from the Heart Center Tissue Bank at Texas Children’s Hospital and from Dr. Marco Ricci (n = 11 HLHS age range: 1–5 days old). Normal human fetal heart protein lysates were purchased from American Life Sciences. Non-diseased fetal heart RNA was purchased from Agilent (pool of 10 hearts from 19–21 weeks old fetuses), Biochain (1 heart from 31 week-old fetus) and Clontech (pool of 5 hearts from 25–40 week old fetuses). RV biopsy samples from neonates with Tetralogy of Fallot (n = 2 age range 2 to 5 months) were obtained from the Heart Center Tissue Bank at Texas Children’s Hospital.

### Study Approval

All human heart tissues used in this study were de-identified and pre-existing, and thus were exempt approved by UTMB Institute Review Board (IRB# 11-087). All animal experiments were conducted in accordance with the National Institute of Health Guidelines for the care and the use of animals approved by the Institutional Animal Care and Use Committee of University of Texas Medical Branch (IAUCUC #1101001).

## Additional Information

**How to cite this article**: Verma, S. K. *et al*. Rbfox2 function in RNA metabolism is impaired in hypoplastic left heart syndrome patient hearts. *Sci. Rep*. **6**, 30896; doi: 10.1038/srep30896 (2016).

## Supplementary Material

Supplementary Information
