# Supplementary material for: Rbfox2 function in RNA metabolism is impaired in hypoplastic left heart syndrome patient hearts
Source: Sci Rep. 2016 Aug 3;6:30896. doi: 10.1038/srep30896 (PMC4971515; doi:10.1038/srep30896)
Supplement: Supplementary Information [file srep30896-s1.pdf]

Supplemental Data:

Rbfox2 function in RNA metabolism is impaired in hypoplastic left heart syndrome patient hearts

Sunil K. Verma[1], Vaibhav Deshmukh[2], Curtis A. Nutter[1], Elizabeth Jaworski[1], Wenhao Jin[3], Lalita Wadhwa[4], Joshua Abata[5], Marco Ricci[6], Joy Lincoln[7,8], James F. Martin[2,9,10], Gene W. Yeo[3],[11], Muge N. Kuyumcu-Martinez*[1,12,13]

[1]Department of Biochemistry and Molecular Biology, University of Texas Medical Branch, Galveston, Texas-77555. Phone: (409) 772-3229, e-mails: skverma@utmb.edu, canutter@utmb.edu, eljawors@utmb.edu

[2]Department of Molecular Physiology and Biophysics, Baylor College of Medicine, Houston, TX 77030 Phone: (713) 798-5931, e-mail: vaibhav.deshmukh@bcm.edu

[3] Department of Physiology, National University of Singapore, Singapore 117597 e-mail: vincenzojin@gmail.com

[4]Division of Congenital Heart Surgery, Baylor College of Medicine, Houston, TX, 77030 Phone: (832) 826-1930, e-mail: lwadhwa@bcm.edu

[5]Pensacola Christian College, 250 Brent Ln, Pensacola, FL 32503 Phone: (850) 478-8496, e-mail: josh.abata@yahoo.com

[6]Department of Surgery, University of New Mexico College of Medicine, Albuquerque, NM, 87131 Phone: (505) 272-6901, e-mail: marcoricci@salud.unm.edu

[7]Center for Cardiovascular and Pulmonary Research and The Heart Center, Nationwide Children's Hospital Research Institute, [8]Department of Pediatrics, The Ohio State University, Columbus, OH 43205 Phone: (614)- 355-5752, e-mail: joy.lincoln@nationwidechildrens.org

[9]Program in Developmental Biology, [10]Department of Medicine, Division of Cardiology, Baylor College of Medicine; Texas Heart Institute, Houston, TX 77030 Phone: (713) 798-5931, e-mail: jfmartin@bcm.edu

[11]Department of Cellular and Molecular Medicine, Stem Cell Program and Institute for Genomic Medicine University of California, San Diego, La Jolla, CA 92093, Phone: (858) 534-9321, e-mails: geneyeo@ucsd.edu

[12]Department of Neuroscience and Cell Biology, [13]Institute for Translational Sciences, University of Texas Medical Branch, Galveston, Texas-77555, USA. Phone: (409) 772-3228, e-mail: nmmartin@utmb.edu

(* the corresponding author)

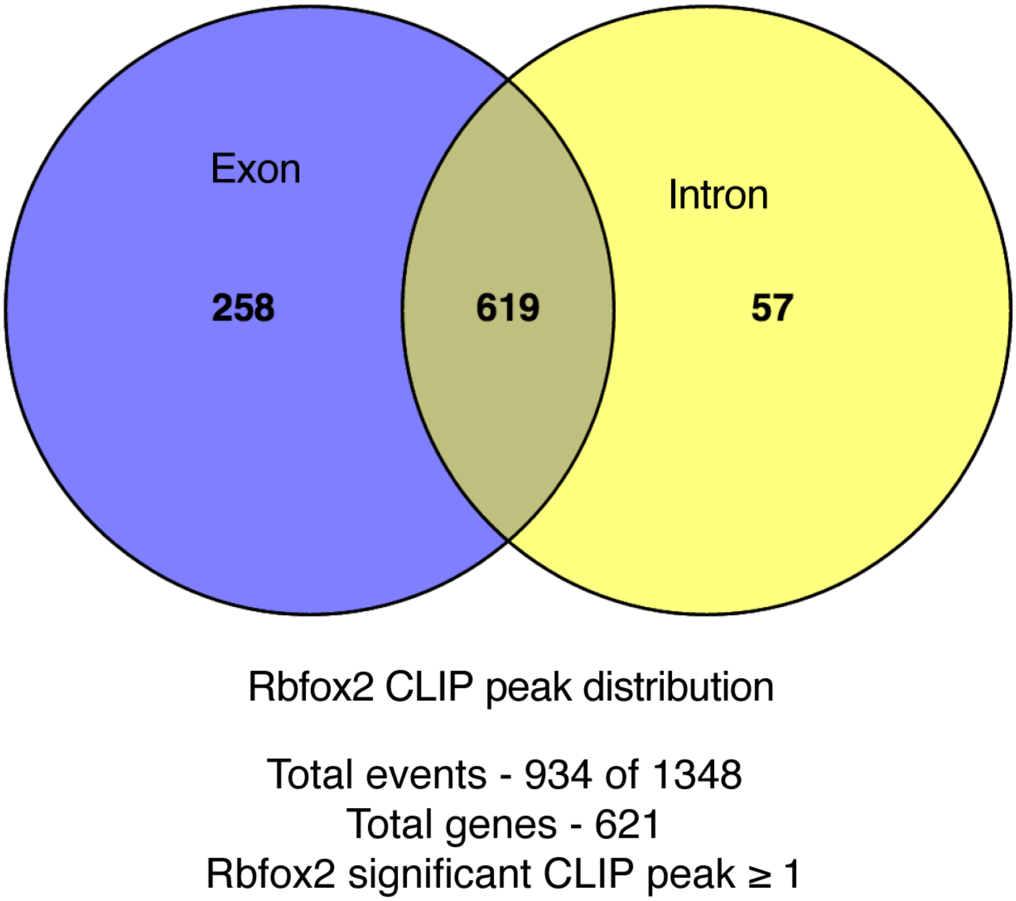

Supplementary Figure 1: Transcripts that are differentially expressed in HLHS have Rbfox2 binding sites. Venn diagram representation of Rbfox2 CLIP-cluster peak distribution in Exon/Intron region of transcripts that are altered in HLHS patient hearts. Venn diagram was created online using venny (http://bioinfogp.cnb.csic.es/tools/venny/index.html).

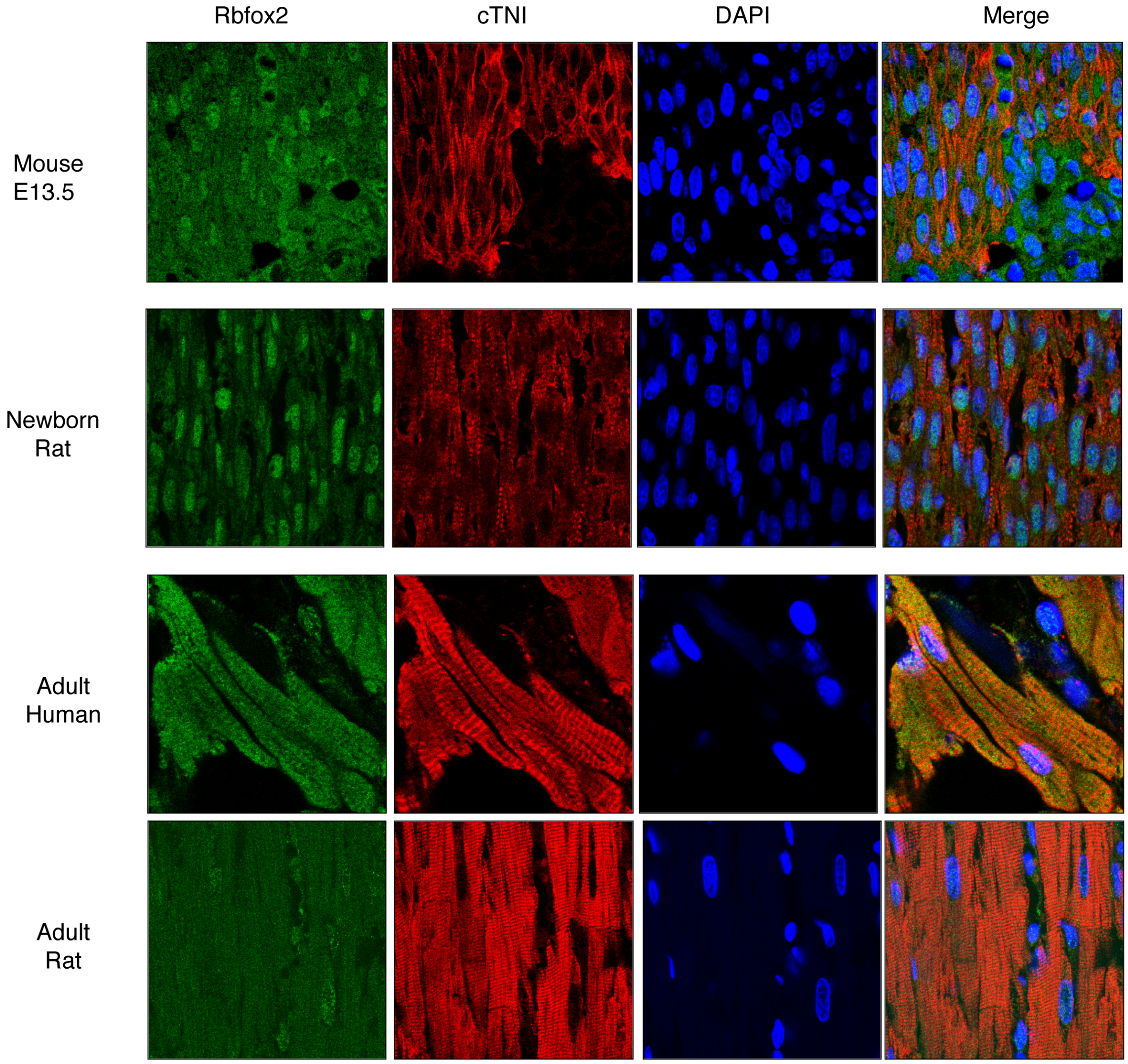

Supplementary Figure 2: **Rbfox2 expression pattern in mouse embryo.** Immunofluorescence of Rbfox2 at embryonic day 13.5 (E13.5) mouse heart, newborn & adult rat heart and adult human heart sagittal section. Cardiomyocyte are marked with cardiac troponin I and nuclei are stained with DAPI.

```
   1 – ATGGAGAAAAAGAAAATGGTAACTCAGGGTAACCAGGAGCCGACAACAACTCCTGACGCA – 60
   1 – M  E  K  K  K  M  V  T  Q  G  N  Q  E  P  T  T  T  P  D  A  – 20

  61 – ATGGTTCAGCCTTTTACTACCATCCCATTTCCACCACCTCCGCAGAATGGAATTCCCACA – 120
  21 – M  V  Q  P  F  T  T  I  P  F  P  P  P  P  Q  N  G  I  P  T  – 40

 121 – GAGTATGGGGTGCCACACACTCAAGACTATGCCGGCCAGACCGGTGAGCATAACCTGACA – 180
  41 – E  Y  G  V  P  H  T  Q  D  Y  A  G  Q  T  G  E  H  N  L  T  – 60

 181 – CTCTACGGAAGTACGCAAGCCCACGGGGAGCAGAGCAGCAACTCACCCAGCACACAAAAT – 240
  61 – L  Y  G  S  T  Q  A  H  G  E  Q  S  S  N  S  P  S  T  Q  N  – 80

 241 – GGATCTCTTACGACAGAAGGTGGAGCACAGACAGACGGCCAGCAGTCACAGACACAAAGT – 300
  81 – G  S  L  T  T  E  G  G  A  Q  T  D  G  Q  Q  S  Q  T  Q  S  – 100

 301 – AGTGAAAATTCAGAGAGTAAATCTACCCCGAAACGGCTGCATGTCTCTAATATTCCTTTC – 360
 101 – S  E  N  S  E  S  K  S  T  P  K  R  L  H  V  S  N  I  P  F  – 120

 361 – CGCTTCCGGGACCCTGACCTCCGGCAGATGTTTGGGCAGTTTGGCAAAATCCTAGATGTA – 420
 121 – R  F  R  D  P  D  L  R  Q  M  F  G  Q  F  G  K  I  L  D  V  – 140

 421 – GAAATAATCTTTAATGAACGTGGCTCTAAGGGATTCGGGTTCGTAACTTTCGAGAATAGT – 480
 141 – E  I  I  F  N  E  R  G  S  K  G  F  G  F  V  T  F  E  N  S  – 160

 481 – GCTGATGCAGACAGGGCCAGGGAGAAATTACACGGCACCGTGGTAGAGGGCCGTAAAATC – 540
 161 – A  D  A  D  R  A  R  E  K  L  H  G  T  V  V  E  G  R  K  I  – 180

 541 – GAGGTGAATAATGCTACAGCACGTGTAATGACCAATAAGAAGATGGTCACACCATATGCA – 600
 181 – E  V  N  N  A  T  A  R  V  M  T  N  K  K  M  V  T  P  Y  A  – 200

 601 – AATGGTTGGAAATTAAGCCCAGTAGTTGGAGCTGTATATGGTCCGGAGTTATATGCAGCA – 660
 201 – N  G  W  K  L  S  P  V  V  G  A  V  Y  G  P  E  L  Y  A  A  – 220

 661 – TCCAGCTTTCAAGCAGATGTGTCCCTAGGCAATGATGCAGCAGTGCCCCTATCAGGAAGA – 720
 221 – S  S  F  Q  A  D  V  S  L  G  N  D  A  A  V  P  L  S  G  R  – 240

 721 – GGGGGTATCAACACTTACATTCCTTTAATCAGTCTCCCTTTAGTTCCTGGCTTCCCTTAC – 780
 241 – G  G  I  N  T  Y  I  P  L  I  S  L  P  L  V  P  G  F  P  Y  – 260

 781 – CCTACTGCAGCCACCACGGCAGCCGCTTTCAGAGGAGCCCATTTGAGGGGCAGAGGGCGG – 840
 261 – P  T  A  A  T  T  A  A  A  F  R  G  A  H  L  R  G  R  G  R  – 280

 841 – ACAGTATATGGTGCAGTCTGAGCGGTACCTCCAACAGCCATCCCCGCCTATCCAGGTGTG – 900
 281 – T  V  Y  G  A  V  *  A  V  P  P  T  A  I  P  A  Y  P  G  V  – 300

 901 – GTTTACCAGGACGGATTTTACGGTGCTGACCTCTATGGTGGATATGCAGCCTACAGATAT – 960
 301 – V  Y  Q  D  G  F  Y  G  A  D  L  Y  G  G  Y  A  A  Y  R  Y  – 320

 961 – GCACAGCCTGCTACTGCAACCGCAGCCACCGCTGCTGCAGCCGCTGCAGCCGCTTACAGT – 1020
 321 – A  Q  P  A  T  A  T  A  A  T  A  A  A  A  A  A  A  A  Y  S  – 340

1021 – GACGGTTATGGCAGGGTGTACACAGCCGACCCCTACCATGCCCTTGCCCCTGCCGCTAGC – 1080
 341 – D  G  Y  G  R  V  Y  T  A  D  P  Y  H  A  L  A  P  A  A  S  – 360

1081 – TATGGAGTTGGCGCTGTGGCGAGTTTATACCGAGGTGGCTACAGCCGATTTGCCCCCTAC – 1140
 361 – Y  G  V  G  A  V  A  S  L  Y  R  G  G  Y  S  R  F  A  P  Y  – 380

1141 – TGA – 1143
 381 – *   – 400
```

Supplementary Figure 3: **Rbfox2 protein is truncated by the *de novo* nonsense mutation identified in patients with HLHS.** Nucleotide sequence and translated protein sequence for human Rbfox2 (NM_001031695.2). Nonsense mutation (C→T) that introduces a stop codon (*) is marked in red.

Supplementary Tables

Supplementary table 1. Functions of representative genes that are differentially expressed in HLHS patients.

**Gene**	**Ensembl ID**	**Function**
Pinin (*Pnn*)	ENSG0000 0100941	*Pnn* encodes for pinin protein that plays a crucial role in small intestinal development by influencing epithelial cell differentiation[1]. Pinin act as splicing co-activator and is involved in mRNA splicing and export in nucleus[2]. Interacts with exon junction complex (EJC) and apoptosis and splicing associated protein (ASAP) complex[3].
Signal peptidase complex subunit 1 (*Spcs1*)	ENSG0000 0114902	SPCS1 is part of microsomal signal peptidase that plays a critical role in the assembly of infectious Hepatitis C virus[4,5].
ATP-dependent RNA helicase DDX39A (*Ddx39*)	ENSG0000 0123136	Ddx39 is an RNA helicase that regulates the switch between cellular proliferation and differentiation in Xenopus[6]. DDX39 is a RNA helicase with pre-mRNA splicing and transport activity[7,8].
DNA replication licensing factor MCM7 (*Mcm7*)	ENSG0000 0166508	MCM7 forms the core of replicative DNA helicase and its polyubiqutylation leads to disassembly of active helicase[9-11].

Phosphorylase b kinase regulatory subunit beta (*Phkb*)	ENSG00000102893	*Phkb* encodes for glycogen phosphorylase kinase that is important for glycogen breakdown and cell growth [12]. Mutation in PHKB gene is involved in autosomally transmitted phosphorylase kinase deficiency of muscle and liver[13].

Supplementary table 2. Primer sequences

**Gene name**	**Forward primer**	**Reverse primer**
Ankyrin2	CGGGGAGGCTCTCCCATCATAC	CTGTTTCTGGGGGTATTTCAG
Rbfox2	GGAAGTTAAGCCCAGTAGTTGG	CCTCCTCTTCCTGACAAGGGC
Rbfox2-DN	CAGTTTGGCAAAATCCTAGATG	GGTGTGACCATCTTCTTATTGG
Pnn	GCGCACACGTAGAGACCTTATCC	GAAGGGTACCCATCAACAAGCC
Phkb	CCAACTACCGGTCTCTTTCCCAC	GAGTGCTCCAGCTCATGGGTCC
Ddx39	GTGCTACCTTGAGCAAAGAGA	AACCCATGCAGCGTCAA
Mcm7	GATGCCACCTATACTTCTGCCCG	GTCCTTTGACATCTCCATTAGCC
Spcs1	CGTGGCTGAACAGTTCGGGTGG	GAACAGGTAACCACTTGAGAGG
Polr2A	CCAGAGTGGATGATTGTCAC	GCGCCGCAGCTGATTGTTGATC
Gapdh	TGGAGTCTACTGGCGTCTT	TGTCATATTTCTCGTGGTTCA

Supplementary References:

1 Joo, J. H. *et al.* Pinin modulates expression of an intestinal homeobox gene, Cdx2, and plays an essential role for small intestinal morphogenesis. *Dev Biol* **345**, 191-203, doi:10.1016/j.ydbio.2010.07.009 (2010).

2 Li, C., Lin, R. I., Lai, M. C., Ouyang, P. & Tarn, W. Y. Nuclear Pnn/DRS protein binds to spliced mRNPs and participates in mRNA processing and export via interaction with RNPS1. *Molecular and cellular biology* **23**, 7363-7376 (2003).

3 Murachelli, A. G., Ebert, J., Basquin, C., Le Hir, H. & Conti, E. The structure of the ASAP core complex reveals the existence of a Pinin-containing PSAP complex. *Nature structural & molecular biology* **19**, 378-386, doi:10.1038/nsmb.2242 (2012).

4 Evans, E. A., Gilmore, R. & Blobel, G. Purification of microsomal signal peptidase as a complex. *Proceedings of the National Academy of Sciences of the United States of America* **83**, 581-585 (1986).

5 Suzuki, R. *et al.* Signal peptidase complex subunit 1 participates in the assembly of hepatitis C virus through an interaction with E2 and NS2. *PLoS pathogens* **9**, e1003589, doi:10.1371/journal.ppat.1003589 (2013).

6 Wilson, J. M. *et al.* RNA helicase Ddx39 is expressed in the developing central nervous system, limb, otic vesicle, branchial arches and facial mesenchyme of Xenopus laevis. *Gene Expr Patterns* **10**, 44-52, doi:10.1016/j.gep.2009.11.001 (2010).

7 Sugiura, T., Sakurai, K. & Nagano, Y. Intracellular characterization of DDX39, a novel growth-associated RNA helicase. *Experimental cell research* **313**, 782-790, doi:10.1016/j.yexcr.2006.11.014 (2007).

8 Sugiura, T., Nagano, Y. & Noguchi, Y. DDX39, upregulated in lung squamous cell cancer, displays RNA helicase activities and promotes cancer cell growth. *Cancer biology & therapy* **6**, 957-964 (2007).

9 Labib, K., Tercero, J. A. & Diffley, J. F. Uninterrupted MCM2-7 function required for DNA replication fork progression. *Science* **288**, 1643-1647 (2000).

10 Moreno, S. P., Bailey, R., Campion, N., Herron, S. & Gambus, A. Polyubiquitylation drives replisome disassembly at the termination of DNA replication. *Science* **346**, 477-481, doi:10.1126/science.1253585 (2014).

11 Maric, M., Maculins, T., De Piccoli, G. & Labib, K. Cdc48 and a ubiquitin ligase drive disassembly of the CMG helicase at the end of DNA replication. *Science* **346**, 1253596, doi:10.1126/science.1253596 (2014).

12 Terashima, M. *et al.* KIAA1199 interacts with glycogen phosphorylase kinase beta-subunit (PHKB) to promote glycogen breakdown and cancer cell survival. *Oncotarget* **5**, 7040-7050, doi:10.18632/oncotarget.2220 (2014).

13 Burwinkel, B. *et al.* Autosomal glycogenosis of liver and muscle due to phosphorylase kinase deficiency is caused by mutations in the phosphorylase kinase beta subunit (PHKB). *Human molecular genetics* **6**, 1109-1115 (1997).
